# Dynamic Navigation Protocol for Direct Sinus Lift and Simultaneous Implant Placement: A Case Report

**DOI:** 10.7759/cureus.53621

**Published:** 2024-02-05

**Authors:** Aditya Dotia, Sahana Selvaganesh, Abhinav R. P., Thiyaneswaran Nesappan

**Affiliations:** 1 Implantology, Saveetha Dental College, Saveetha Institute of Medical and Technical Sciences, Chennai, IND; 2 Oral Surgery, Saveetha Dental College, Saveetha Institute of Medical and Technical Sciences, Chennai, IND; 3 Prosthodontics, Saveetha Dental College, Saveetha Institute of Medical and Technical Sciences, Chennai, IND

**Keywords:** navident, sinus floor elevation, maxillary sinus, dynamic navigation, direct sinus lift

## Abstract

Aim: This study aims to evaluate the accuracy associated with the use of a dynamic navigation system for the lateral window opening for a direct sinus floor elevation (SFE) procedure with simultaneous implant placement.

Materials and methods: A female patient, aged 27 years, reported to the Department of Implantology seeking treatment for her lost tooth. On radiographic examination, the residual alveolar ridge height was 6 mm in the 26 (left upper first molar) region. For the implant placement, the case was planned to be carried out under dynamic navigation (Navident, Claronav, Canada). To make the lateral window accessible to the sinus floor, an implant trajectory resembling the required window dimensions and prosthetic implant position was planned. Post-surgery cone beam computed tomography (CBCT) was taken to assess the accuracy of the lateral window and implant trajectories using Evalunav (Navident, Claronav, Canada) analysis with dynamic navigation software.

Results: There was improved accuracy of the lateral window opening, and the visualization of the lateral window was maintained in real-time throughout the procedure, which was advantageous to eliminate the tearing of the thin sinus membrane. The deviations found in the trajectory of the lateral window in comparison between the planning and post-procedure were: (a) entry was deviated by 2.83 mm; (b) the apex was deviated by 2.52 mm; (c) vertically, the apex was deviated by 0.29 mm; and (d) there was an 8.93° deviation in the angulation of the trajectory. The implant that was placed simultaneously with the SFE’s accuracy was in comparison with the position that was planned: (a) entry was deviated by 0.03 mm, (b) the apex was deviated by 0.82 mm, (c) vertically, the apex was deviated by 0.82 mm, and (d) there was a 0° deviation in the angulation of the trajectory.

Conclusion: Dynamic navigation technology can help overcome complications associated with direct sinus lift procedures by providing highly accurate and precise planning and execution of the surgical procedure. This can lead to improved implant stability and a reduced risk of complications.

## Introduction

A sinus floor elevation (SFE) procedure, or sinus augmentation, is a surgical procedure performed to gain bone height in the posterior maxilla in the area of the molars and premolars. This is necessary when there is insufficient bone height in the upper jaw, which can be caused by factors such as tooth loss, periodontitis, or bone loss due to aging. The SFE procedure involves lifting the sinus membrane and placing bone graft material into the space created between the floor of the sinus and the sinus cavity. This creates additional bone height, allowing for the successful placement of dental implants in the upper jaw and improving primary stability [1].

The approach to the sinus can be direct or indirect. In direct SFE, also known as the lateral window technique, the sinus lining is elevated by direct vision by making a lateral window to gain access to the sinus floor lining to raise it and place the bone graft. According to a systematic review and meta-analysis published by Antonoglou et al., the dental implant success rate in areas where sinus lift procedures were performed ranges from 92.3% to 98.6% [2]. This indicates that the sinus lift procedure is viable for patients with insufficient bone height in the posterior maxilla who wish to undergo dental implant placement [3].

Though SFE procedures have a high success rate, potential complications can occur during the surgical procedure. The most common is Schneiderian membrane perforation [4-5]. This can cause complications like graft migration into the sinus and implant failure. Keeping this in mind, it is important for dental professionals to carefully assess the patient's anatomy and perform the procedure with precision to minimize the risk of complications [6]. Close monitoring and follow-up with the patient are crucial to detecting and managing any potential complications [7].

Dynamic navigation in dental implantology can help overcome complications associated with direct sinus lift procedures. Dynamic navigation is a relatively new technology increasingly used in dental implantology to improve accuracy and safety during surgical procedures, including direct sinus lift procedures [2,8]. This technology uses real-time imaging and computer-guided navigation to allow for the precise and predictable placement of dental implants [9]. The working principle is triangulation, aided by optical sensors that are illuminated by the LED light panel. The optical sensors are placed in the triangle zone, namely, the patient's jaw (jaw tracker) and the operator's dominant arm carrying the handpiece with the drill tag. This is achieved by precisely planning the implant prior to the surgical appointment with the available radiographic aid. The Navident system will guide the dentist's hand movements and ensure precise implant placement. The system provides real-time feedback to the dentist, allowing for adjustments to be made as needed. Additionally, dynamic navigation can reduce surgical time and trauma to the surrounding tissue, decreasing the risk of complications such as infection and postoperative pain [9]. Implantology procedures should be carried out with enhanced precision, and the healing of the soft tissues is of the utmost importance [9-11]. This is vital for assessing the implant's health and choosing abutments for the long-term success of the implants [12].

Dynamic navigation can also help in educating the patient, as the patient will be made comfortable and transparency can be maintained throughout the procedure, which can reduce the overall anxiety of the patient [13]. For improved accuracy, dynamic navigation can also be coupled with static navigation systems, which can yield effective results [14-15].

According to a systematic review and meta-analysis by Pellegrino et al., dynamic navigation in dental implant surgery has been associated with improved implant survival rates and a reduced risk of implant-related complications. The navigation group had decreased implant placement errors (p<0.01) and similar accuracy (p≥0.05) compared to the static approach. The pooled prevalence of failures was 1% (95% CI: 0.00% to 2%) [16]. In this study, we try to create a workflow to use Navident to guide the surgeon while performing a SFE with immediate implantation using a lateral window technique.

## Case presentation

Patient selection

This study was conducted at the Department of Implantology, Saveetha Dental College and Hospital, Chennai, India. The following criteria were followed to include the patient for working under dynamic navigation: patients eligible for inclusion were as follows: (A) patients desiring posterior maxillary implants with 4-6 mm residual alveolar ridge height (indicating the need for direct SFE); (B) individuals with maximum mouth opening (40 mm) for Physiodispenser handpiece drill orientation; (C) for whom a preoperative digital visual implant plan was prepared; and (D) people in good health without any systemic diseases that are contraindicated for implant placement [8].

Patients were excluded from this study if they exhibited reduced mouth opening, irradiation of the jaws, bad oral hygiene, heavy smoking, bisphosphonate treatment, and uncontrolled diabetes, with the potential to impact the implant surgery procedure or the use of a dynamic navigation system.

**Figure 1:**
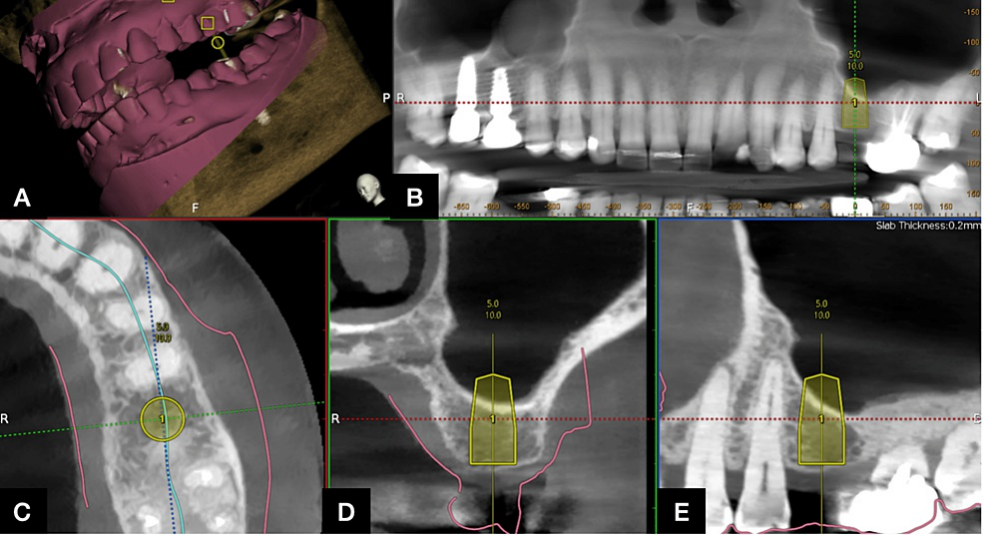
Implant planning under the dynamic navigation software (Navident) (A) Represents the cone beam computed tomography (CBCT) data and the STL files. (B) Implant planning 26 OPG view. (C) Occlusal view of implant position. (D) Bucco-lingual placement of the virtual implant. (E) Mesio-distal placement of the virtual implant

Based on the eligibility criteria mentioned above, a female patient, aged 27 years, who presented at Saveetha Dental Hospital was selected for this procedure. The patient required implant placement in 26 regions (Figure 1) with a residual alveolar ridge height of 6 mm.

Preoperative preparation

A CBCT of the patient was derived using a Carestream CBCT machine (CS9600) and added to the dynamic navigation software to plan the spatial location of the implant and the lateral window. The dynamic navigation software used for the study was Navident version 3.0 (Navident, Claronav, Canada). The stereolithography (STL) file of the patient's upper jaw was also merged through the Navident software, and optimal implant diameter and length were selected. The residual bone height available before the augmentation was 6 mm. The implant location and orientation were then selected, and the required SFE was determined. An implant trajectory of 10 mm length and 5 mm diameter was selected in this case (Figure 1).

**Figure 2:**
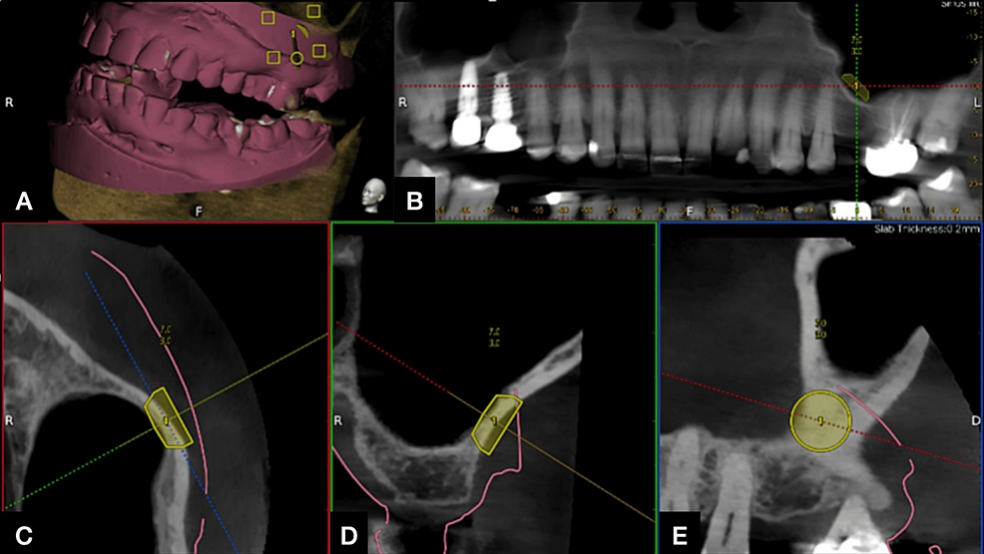
Lateral window trajectory planning under the dynamic navigation software (Navident) (A) Cone beam computed tomography (CBCT) data and stereolithography (STL) files merged into the software. (B) Lateral window opening planning in the orthopantomogram (OPG) view. (C, D, E) Lateral window planning in the occlusal, bucco-lingual, and mesio-distal view

Since the Navident software doesn't have a dedicated function to make a lateral window, the implant trajectory option was selected, and the shape, location, and orientation of the implant trajectory were modified to make a lateral window to access the maxillary sinus. A trajectory of 7 mm diameter and 3 mm depth was planned (Figure 2). This trajectory was later used as the path for the trephine bur for the lateral wall removal and direct sinus lift procedure.

Surgical procedure

**Figure 3:**
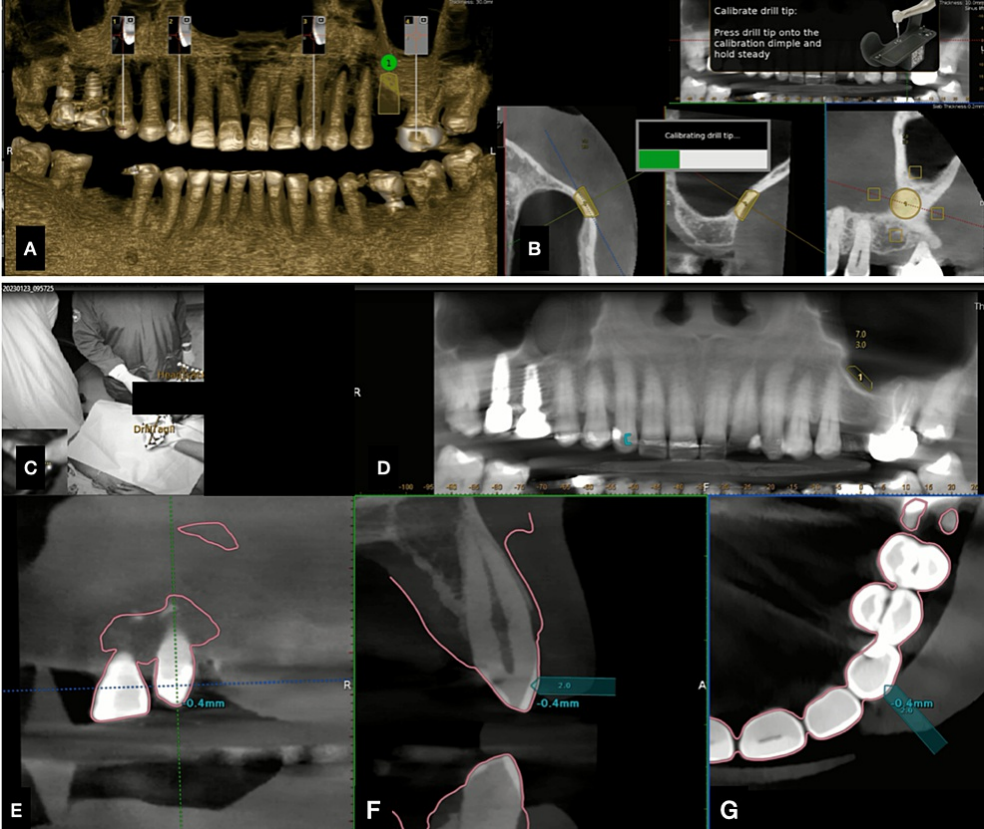
Working with dynamic navigation (A) Points in the cone beam computed tomography (CBCT) for trace registration. (B) Calibration of the drill tip. (C) Drill tag and jaw tracker detected by the computer. (D) Accuracy checking of the trace registration using the drill tip on the cusp of the index tooth. (E) Accuracy checking with the drill tip. (F) Accuracy check with the drill tip of the adjacent tooth. (G) Occlusal region accuracy check of the calibrated data

Two surgeons performed the procedure. One surgeon with two years of experience in implant placement was guided by the other with five years of experience. The first step was to place the head tracker in place to determine the relative position of the patient as compared to the stereo camera of the dynamic navigation unit. A trace registration device was used in the reference teeth for the camera to track the jaw in real-time, and for improved accuracy, this step is referred to as trace registration (Figure 3). Following the registration of the particular jaw with the navigation unit, the handpiece was attached, and each drill calibration step was carried out.

**Figure 4:**
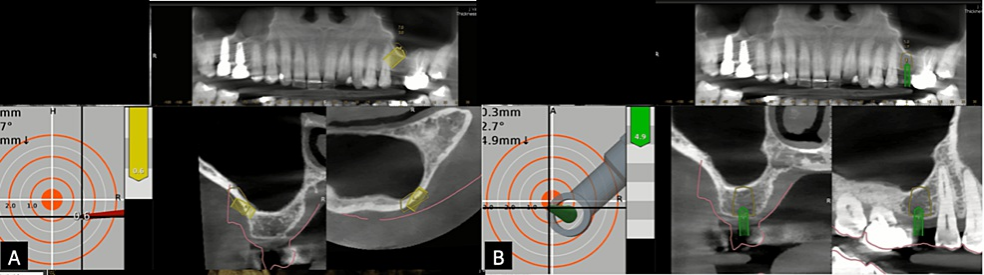
Drilling under dynamic navigation (A) Computer feedback of the lateral window drilling with the trephine bur. (B) Precision of drilling in the planned implant site drilling under dynamic navigation

A transcrestal incision with releasing incisions was then given. The trephine bur of diameter 7 mm was depth calibrated for the lateral window cut. Then, the lateral window was made using the real-time dynamic guiding system. The sinus lining was now gently separated from the sinus floor. Once the SFE procedure was done, the osteotomy of the implant was made under real-time guidance (Figure 4), with sequential drills being calibrated with the Navident unit.

**Figure 5:**
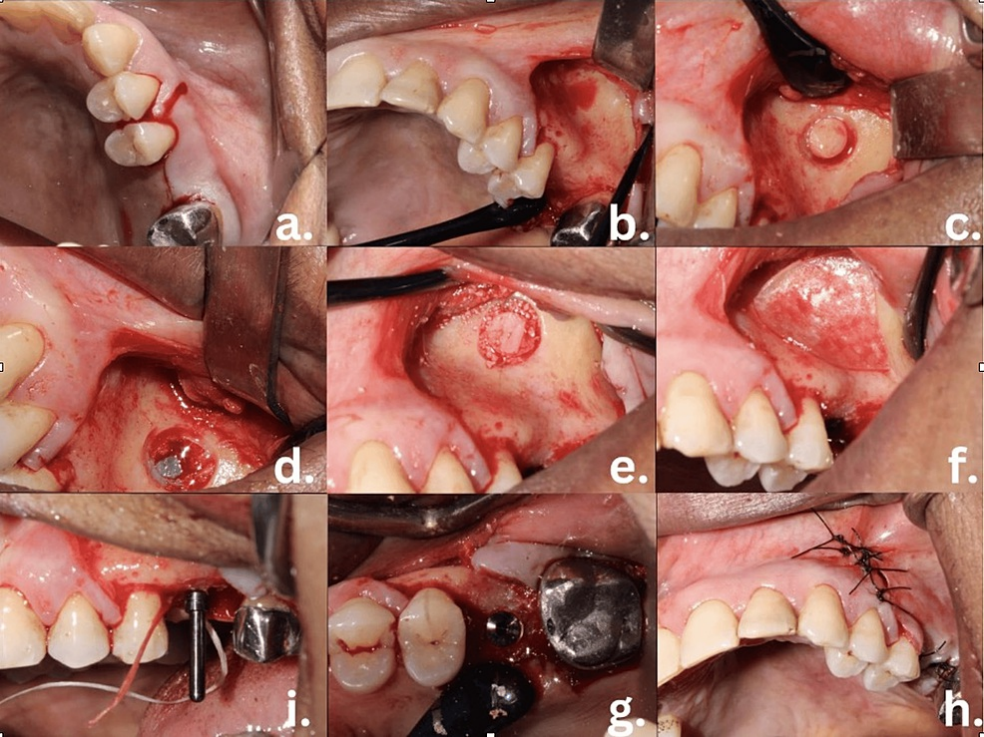
Surgical procedure and implant placement (A) Incision. (B) Flap elevation. (C) Lateral window made using trephine bur under the guidance of Navident. (D) Lateral window opening to expose sinus lining. (E) Sinus lining separated and elevated from the floor of the sinus, bone graft placed. (F) Membrane placed to cover the lateral window opening. (J) PID for implant placement. (G) Implant placement at the site. (H) Suturing of the site

Bio-Oss bone graft (Geistlich Pharma North America, Inc., NJ, USA) was then condensed under the sinus floor lining to promote bone growth. The implant was then screwed into the osteotomy, further condensing the bone graft (Figure 5) [9].

**Figure 6:**
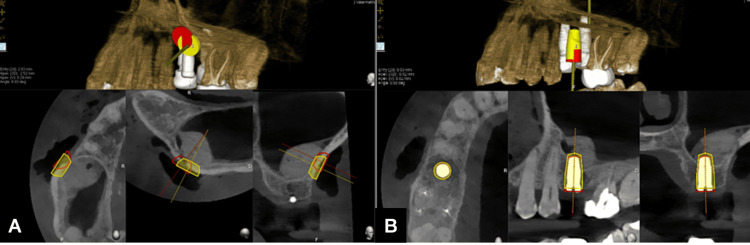
Evalunav analysis of the lateral window and the implant placement (A) Evalunav analysis of the lateral window. (B) Evalunav analysis of the implant placement

A CBCT was taken of the patient immediately after surgery, and antibiotics and analgesics were prescribed for five days. The patient was recalled after seven days for suture removal and a case review. A blinded specialist used Evalunav (Navident, Claronav, Canada) dynamic navigation accuracy verification software to measure discrepancies between the planned preoperative and postoperative implant placements by merging the pre-planning CBCT and post-implant placement CBCT (Figure 6). The sinus space was checked for graft extrusion or rupture.

After analyzing the post-CBCT, the deviations found in the trajectory of the lateral window were as follows: (a) Entry was deviated by 2.83 mm, (b) the apex was deviated by 2.52 mm, (c) vertically, the apex was deviated by 0.29 mm, and (d) there was an 8.93° deviation in the angulation of the trajectory. The deviations found in the trajectory of the implant were as follows: (a) Entry was deviated by 0.03 mm, (b) the apex was deviated by 0.82 mm, (c) vertically, the apex was deviated by 0.82 mm, and (d) there was an 0° deviation in the angulation of the trajectory (Table 1).

**Table 1:** Results of the Evalunav analysis Assessment of the accuracy between the planned and postoperative positions of (A) implant placement and (B) lateral window opening Entry (2D): deviation in mm at the planned and placed implant position, Entry (3D): deviation in angulation, Apex: deviation of the apex in mm, Angle: angular deviation in degrees

S. no.	Entry (2D) (mm)	Entry (3D) (mm)	Apex (V) (mm)	Angle (deg)
Implant placement	0.03	0.82	0.82	0.00
Lateral window opening	2.83	2.52	0.29	8.93

## Discussion

The maxillary sinus is lined by a membrane called the Schneiderian membrane. This membrane is extremely thin, averaging 0.4 mm to 1.5 mm in most individuals [17,18]. Furthermore, several anatomic anomalies can further complicate a sinus lift procedure. A septum within the sinus is a common anomaly that can create trouble while performing an SFE. A study reported that 21.58% of the population showed a septa within the sinus floor [19]. Other anomalies, like a polyp within the sinus oblique dipping of the sinus floor, can also complicate the procedure and make the proper placement of the lateral window more vital [20]. The risk of sinus membrane rupture during a direct sinus lift can vary depending on several factors, including the sinus's anatomy, the surgeon's experience and skill, and the technique used. Proper preoperative assessment, including radiographic imaging such as CBCT, can help evaluate the thickness and integrity of the sinus membrane and aid in treatment planning [17,18,20].

To mitigate the risk of sinus membrane perforation, surgeons typically employ careful surgical techniques and instruments designed for sinus lift procedures. Proper instrumentation, such as specially designed osteotomes or piezoelectric devices, can help minimize the risk of damaging the sinus membrane during bone preparation. Additionally, the surgeon's experience and skill are crucial in reducing the chances of membrane rupture. Experienced surgeons who are familiar with the anatomy of the sinus and have performed numerous sinus lift procedures are generally more adept at minimizing complications [21]. Regarding the armamentarium, the choice between the piezo tome and trephine bur depends on various factors, including the surgeon's preference, the patient's anatomy, and the case's complexity [21]. Some surgeons may prefer the Piezotome for its precision and safety, especially in cases with thin sinus walls or near the sinus membrane. On the other hand, the trephine bur may be suitable when a smaller access point is desired, or in cases where a significant amount of bone removal is required, post-operative pain should also be considered [17,22].

Dynamic navigation can be a game-changer in implant surgery, which utilizes real-time computer guidance for precise implant placement. The accuracy of it can be influenced by various factors. Preoperatively, the stage is set by CBCT image quality, which can stumble under the influence of hardware, software, and even the surgeon's touch. A misplaced registration device can hamper accuracy. Regular calibration acts as a meticulous rehearsal, ensuring the device works flawlessly. Lack of familiarity with the technique can lead to poor performance, impacting accuracy. The mouth opening acts as a set limitation, potentially restricting access to the posterior maxilla and disrupting the flow. Finally, the bone itself plays a crucial role, with its density and anatomy dictating the placement of the implant. By considering these factors, surgeons can ensure dynamic navigation and perform a flawless procedure, leading to optimal outcomes for their patients.

Wu et al., in their study. assess the accuracy of the dynamic navigation system for transcrestal sinus lift procedures, reported the accuracy of angular deviation, entry point horizontal deviation, and apical point horizontal deviation between the planned and actual implant placement were 3.656 ± 1.665°, 1.073 ± 0.686 mm, and 1.086 ± 0.667 mm, respectively [23]. Case reports by Su et al. [24] have platform deviations of 1.60 and 2.24 mm, apex deviations of 1.46 and 2.13 mm, and angular deviations of 1.18° and 5.50°. This is comparable to the present study, where there is 0° angular deviation and <1 mm deviations in mesio-distal, bucco-lingual, and apico-coronal aspects pertaining to implant placement involving the sinus.

This particular case used trephine burs for the lateral window opening. The methodology of using a trephine bur for calibration and opening the lateral window has not been attempted or researched prior. The provision for using a piezo handpiece is also viable under dynamic navigation, just that the drill tags have to be ideally attached to the piezo handpiece, and there was an 8.93° deviation in the angulation of the trajectory, which is acceptable. Dynamic navigation through its advanced imaging and tracking ability helps create a virtual three-dimensional map of the patient's mouth using the CBCT loaded into the software. It acts like a GPS tracking mechanism, helping the surgeon maintain the safety of the anatomical structures and providing improved control. This helps the surgeon navigate the patient's anatomy accurately and precisely, reducing the risk of error [23,25,26]. Providing real-time feedback minimizes the chances of damaging the sinus membrane.

Furthermore, it makes the outcome more predictable by narrowing the gap between virtual planning and actual treatment. Randomized control trials with an improved sample size can provide more information on the success and survival rates of implants placed in the sinus under dynamic navigation. One disadvantage of dynamic navigation is that soft tissue cannot be properly controlled without the proper information. The surgeon has to possess an improved haptic tactile feedback mechanism for identification and sinus membrane thickness to differentiate between soft and hard tissue using a dynamic navigation system attached to the ideal physio dispenser handpiece. With drilling without actually orienting or looking at the surgical site but rather looking at the screen, the operating surgeon's hand has to have the tactile sense to differentiate between the hard and soft tissues. Dynamic navigation has a steep learning curve and depends highly on the information's accuracy. Regular calibrations of high-quality CBCTs are a must while using dynamic navigation [18].

## Conclusions

Dynamic navigation technology can help overcome complications associated with direct sinus lift procedures by providing highly accurate and precise planning and execution of the surgical procedure. This can lead to improved implant stability and a reduced risk of complications. However, it is important to note that dynamic navigation is not a replacement for careful surgical planning and execution by a skilled and experienced dental professional.
